# Magic Circle

**DOI:** 10.1177/2041669518770691

**Published:** 2018-05-07

**Authors:** Jan Koenderink, Andrea van Doorn, Johan Wagemans

**Affiliations:** Laboratory of Experimental Psychology, University of Leuven (KU Leuven), Leuven, Belgium; Justus Liebig Universität, Giessen, Germany; Utrecht University, Utrecht, Netherlands; Justus Liebig Universität, Giessen, Germany; 8125 Utrecht University, Utrecht, Netherlands; University of Leuven (KU Leuven), Leuven, Belgium

**Keywords:** panoramic vision, visual space, pictorial space, horizon

## Abstract

Full-horizon cylindrical projections of the optic array are in common use. One wonders whether the public actually profits from such pictorial information, since the space behind one’s back does not exist in visual awareness. In an experiment, a test image included six persons located at the corners of an irregular hexagon centred at the camera. Two persons faced the camera, two turned their back to the camera and two others faced a direction at right angles to the camera. The distances to the camera were unequal and varied from 1 to 2 m. Participants were asked to draw a ground plan of the perceived configuration, including actors and camera, on the basis of viewing the picture. As with any picture there exist many possible interpretations, the ambiguity grows even more when the angular scope of the picture is unknown. Almost all naïve viewers parse this planispheric (Mercator) representation so as to have the whole scene in front of them, with the actors standing in a circle, facing each other. They take the viewpoint to be *outside* the circle. Only a few placed the viewpoint *inside* the circle, which is indeed another reasonable interpretation (in this case the actual one).

## Introduction

Full optic array (Burton, 1945; Gibson, 1950; Koenderink, Albertazzi, et al., 2010; Reid, 1819) panoramic cameras are becoming increasingly available and popular.

Naïve observers have considerable problems to deal with very wide-angle (but less than 180°) images (Attneave & Farrar, 1977; Koenderink, van Doorn, de Ridder, & Oomes, 2010; Phillips & Voshell, 2009).

In the common full-horizon, cylindrical projections (e.g., the popular equirectangular or equidistant rectangular, French: *plate carrée*, German: *quadratische Plattkarte*; Snyder, 1993) observers routinely ignore the periodic structure (Koenderink & van Doorn, 2017). This happens even when interactive panning is available. In popular ‘rolling ball’ dynamic, interactive renderings one routinely confuses the interior and exterior orientations (Koenderink & van Doorn, in press). The problems are due to a lack of intuitive grasp of the topology, the fact that the horizon is a closed curve and that the viewing sphere is viewed from the inside, whereas rolling ball graphics show the outside.

One might say that the full horizon is a ‘magic circle’ that – in depictions – cannot be entered by the observer (Buckland, 2002). Something similar applies to the full viewing sphere. In this contribution we explore that notion in more – also quantitative – detail.

In this article, the emphasis is on static images of the familiar ‘postcard’ variety, that is, roughly A5-size viewed informally at normal reading distance. The most popular static representations are cylindrical projections with the horizon represented as a (straight) horizontal line. Various cylindrical projections, in which the verticals are rendered as vertical straight lines, are in common use. In this experiment the Mercator representation, a planispheric, conformal map is used (Mercator, 1569). Its conformal property is nice whereas global deformations are limited if the elevations are not too close to either zenith or nadir.^1^

Such a cylindrical full-horizon rendering yields a rectangular picture. Its aspect ratio depends on the range of elevations. So we skip two ‘polar-caps’ centred at zenith and nadir. In practice, we decided on a desired aspect ratio (postcard format) and let that constrain the extreme elevations. At first blush such a picture appears as a regular postcard.

In previous experiments (Koenderink & van Doorn, 2017, in press) we showed that human visual awareness is simply unable to deal with such renderings. In these experiments we tried hard to make the visual task as simple as possible for the observers. In contradistinction, in the present experiment we intentionally set up a spatial configuration in such a way as to ‘fool’ the observer: By having the pictorial content conform to a familiar scene we enforce the ‘generic postcard’ situational awareness and thus silently suggest an inappropriate scope. It is indeed not hard to design spatial configurations that will almost certainly be perceived in some specific manner by the overwhelming majority of naïve observers. This is just intuition; there is no science of the matter. This ability might well find applications in – for instance – the movie business.

Well-documented misinterpretations (Koenderink, van Doorn et al., 2010; Koenderink & van Doorn, 2017) are due to the fact that a postcard type of image is routinely interpreted as the rendering of a ‘normal’ field of view, roughly spanning 40deg to 80deg. In the case of regular photographs the actual field of view may range from a few degrees (‘tele’ shots) up to 150deg (extreme ‘wide-angle’) view. Observers typically do not know the actual field of view and interpret any image in terms of their implicit ‘normal’ view (Koenderink, van Doorn et al., 2010), sometimes leading to apparent ‘deformations’ seen in perfect perspective renderings (Brandt, 1961).

In the case of extremely panoramic (horizontal field of view exceeding 180deg) images there is an additional complication in that they contain content that would be behind the observer’s back. In generic visual awareness the space behind the back does not exist in a visual sense (Phillips & Voshell, 2009). We have already shown that this yields completely novel types of characteristic errors in pictorial perception (Koenderink & van Doorn, 2017) that go beyond the usual ‘Ames room-type’ (Ittleson, 1952) effects.

The effects can often be predicted at least semiquantitatively from simple models of the structure of visual space, as we have shown for extreme wide-angle views (Koenderink, van Doorn et al., 2010). To extend this to cases that include the space behind the back is such an extreme extrapolation as to be a shot in the dark. As we show here, it actually works quite well. Since most readers will be unfamiliar with the log-polar visual space model (Koenderink & van Doorn, 2008), we succinctly summarise the basics in the following.

## Methods

The aim was to design a spatial configuration in such a way that naïve observers would be grossly deceived in their ‘reading’ of the picture. We aim at categorical mistakes, rather than mere (even if large) quantitative errors. The aim was to produce a planispherical map that could readily be confused with a regular photograph showing a ‘normal’ scope of 40° to 80° instead of the full 360°.

### Design of the Configuration

One would like to apply the Ames room technique (Ittleson, 1952) to create optically equivalent configurations. However, in the case of full-horizon renderings this is evidently not possible, because the Ames room technique conserves the identity of ‘visual rays’ and merely shifts positions on these rays. For the present application one also needs to *change the visual directions*. This can be done using a general model of visual space (Koenderink & van Doorn, 2008).

We have shown the power of this model for the case of extreme wide-angle (e.g., fish-eye lens) views (Koenderink, van Doorn et al., 2010; Koenderink & van Doorn, 2017). Effects are huge, observers committing misjudgements of the spatial attitude of pictorial objects exceeding 100deg. (Perhaps surprisingly, the misjudgements are as extreme as that, although the textbooks do not mention them.) This still applies to the space in front of the camera though.

Here we go categorically beyond that, including the space ‘behind the camera’. There exist no observations on such cases, so we are in no position to predict how this will work out. The difference is indeed categorical because human observers are physiologically limited to the optical space in front of them (Helmholtz, 1896). Going beyond that even introduces changes of a topological nature, so all bets are off. We aim at a design that might be interpreted in two, mutually very different, ways: In one interpretation, the camera is at the centre of a group of people and in the other interpretation, the camera is outside the group. The mutual spatial attitudes in the two interpretations are also very different. Here is the design:

Consider a group of six persons facing each other, positioned on the vertices of a regular hexagon. In the case of a ‘normal’ photograph, the camera will be outside the hexagon. Usually, the distance of the camera to the group will be large with respect to the diameter of the hexagon, on an axis of bilateral symmetry, with three persons on each side of the main camera direction. Then, two persons will roughly face the camera, two will turn their back to the camera and two will be seen in profile, facing the camera axis. The persons facing the camera will be at the largest, those turning their backs to the camera at the shortest and those seen in profile at some intermediate distance from the camera.

**Figure 1.**
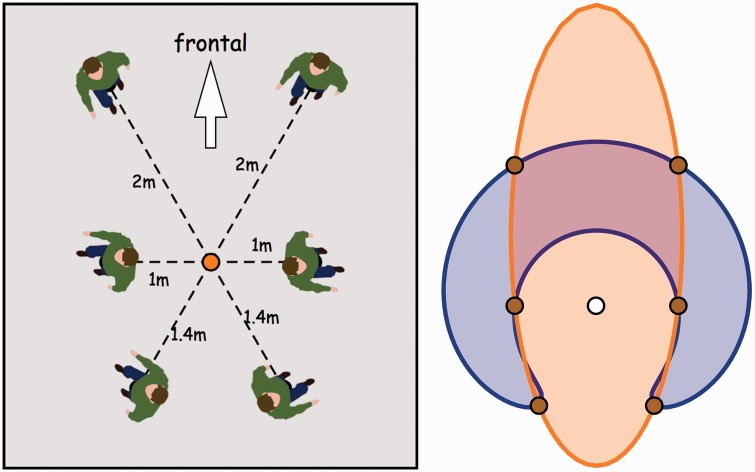
*Left:* Ground plan for the photograph used in the experiment. The camera is at the central orange disk. Notice that the actors are at distances of either 1 m, 141 cm ($\sqrt{2}$m) or 2 m from the camera, in directions at $60^{\circ }$ intervals. The formal ‘frontal’ direction has been added for easy reference. *Right:* Here the camera position is the white point, the points of the hexagonal configuration are indicated with the brown points. The boundary of the orange region is an ellipse passing through the points, the boundary of the blue region (to be discussed in detail later) also passes through the points. The camera is *inside* the orange region, but *outside* the blue region. This is the topological relation that we are addressing in this study. The two regions are related through a remapping of visual directions, they are optically *fully equivalent interpretations*. Either region is an okay interpretation of the picture.

For a full 360° panoramic view, one obtains a configuration in the ground plan shown in Figure 1 left. (Figure 1 right shows two areas defined by the points of the hexagonal configuration that will be discussed later in the article.) We picked a minimum distance of 1 m in order to obtain an angular height of about 60deg, roughly equal to the spacing along the horizon. In Mercator projection, this will then yield a reasonable (postcard variety) aspect ratio.

**Figure 2.**
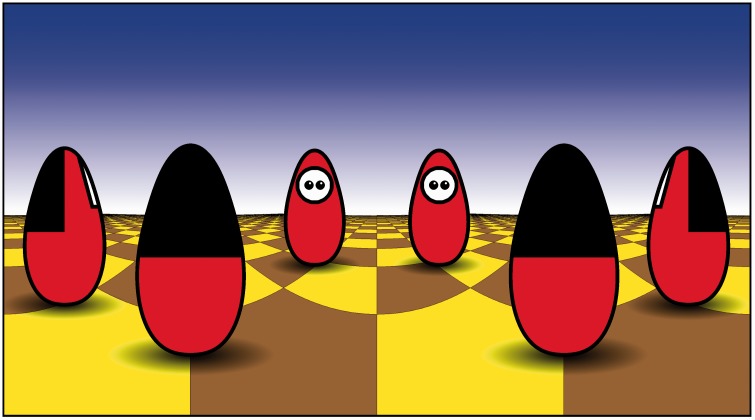
An artificial scene. The ‘actors’ (mutually identical Daruma dolls) are placed on an infinite floor with regular square tiling. By noticing the vanishing points of the grout lines at the horizon, you easily figure out the extent of the field of view. By following the curved grout lines (which are actually straight lines), you may figure out the actual orientation of the figures with respect to each other. One should be able to work out the actual layout, all the necessary cues are explicitly present. Few observers can do this *a prima vista though*.

In Figure 2 we show a simulation on a regular tiled floor with square tilings in Mercator projection, thus fully exhibiting the actual layout. Yet, what we see (as colleagues that were shown the image) is a group of actors arranged in a circle, facing each other. This suggests that observers are generally unable to make good use of such images, even if they know what they are looking at. We already know that from experience with full-horizon images in various settings (urban scenes, natural landscapes and indoor scenes). Given the growing use of full-horizon images, it is important to gain some notion as to the degree of veridicality of their immediate impressions.

In this report we test an actual image on a large number of observers. This allows us to arrive at expectations of the kind of configuration observers are likely to report. Such expectations depend on two generic principles, namely: *firstly*, the spatial attitude of visual objects is judged with respect to the local line of sight, and *secondly*, depicted scenes are taken to be located in a half-space in front of the viewpoint. The scene designed here can be interpreted in an infinity of ways, although two might be termed ‘cardinal’. In a later section these cardinal views are compared in some detail. In the experiment we attempt to quantify the probability of obtaining one or the other cardinal view. As argued earlier, we expressly set up the scene to suggest one of these. The depiction of the full-horizon scene is typical for the images that are already widely used by the general public.

The configuration was set out in the Leuven city park. The locations of the actors were previously constructed with the help of a measuring tape and indicated by hammering tent pegs in the ground.

**Figure 3.**
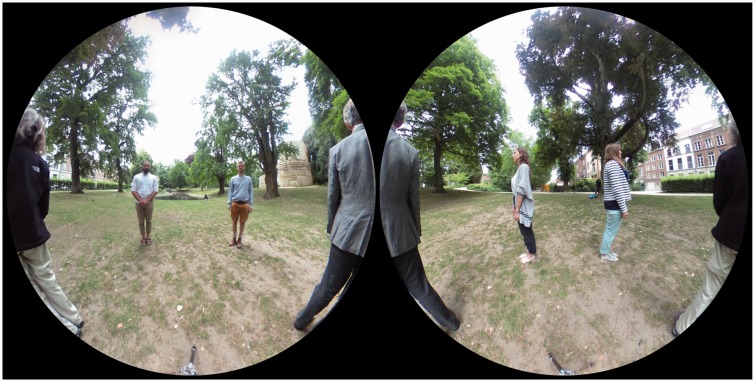
A stereographic map of what is ‘in front’ (left) and what is ‘at the back’ (right) of the camera. The scene layout is shown in Figure 1 left. The zenith and nadir are represented at top and bottom points in either picture. The circular outlines of the pictures coincide. Like the Mercator map, the stereographic map is conformal, but not area true.

In Figure 3, it is shown what was in front and what at the back of the camera. Here the ‘frontal direction’ has only a formal meaning (indicated in Figure 1 left), since the camera itself has no preferred ‘viewing direction’ but is fully isotropic. The stereographic projections each show a full half-space. Notice that their (circular) outlines coincide.

The photograph destined to be used as the stimulus was taken with a Ricoh Theta S panoramic camera from roughly average navel or breast-height. Thus the horizon will roughly bisect the vertical extent of the figures, which is desirable in order to balance the magnification increase with visual height of the Mercator projection.

**Figure 4.**
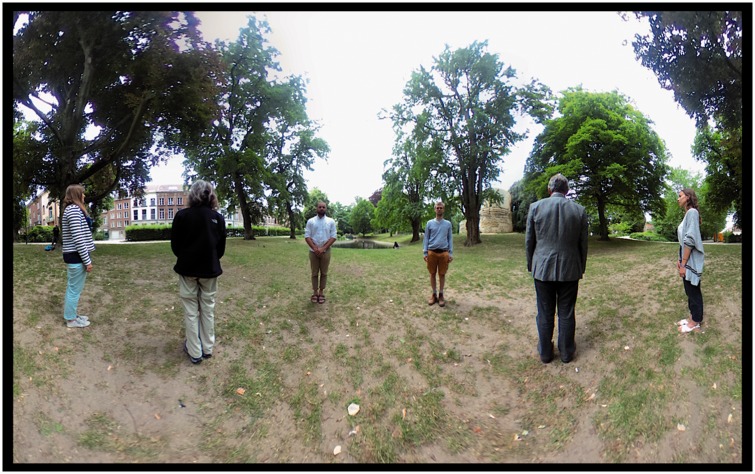
The photograph used as stimulus in the experiment. The ground plan of the physical configuration is shown in Figure 1 left. The projection is Mercator, with the horizon at the half-height of the picture. Notice that the actors are equally spaced on the horizon. The pictorial vertical extent of the actors varies by about a factor of two (the persons were not of exactly the same size). The actors are seen in either anterior, posterior or lateral (profile) view. The location of the feet is another cue to distance: since the terrain was roughly horizontal, higher in the picture plane indicates greater distance from the camera. The picture has a periodic topology in the sense that the left and right edges show the same direction of view (purely posterior). Zenith and nadir are at ±∞, upwards and downwards in the picture plane, they have been arbitrarily cropped. The horizon roughly cuts the actors in half, thus indicating the height of the camera.

The camera was remote controlled by means of an iPhone, so the photographer does not appear in the photograph. The Mercator map (see Figure 4) was computed by way of a simple program written in Processing 3. It can hardly be distinguished from the raw camera image (an equirectangular map).

#### Definition of coordinates and angles

**Figure 5.**
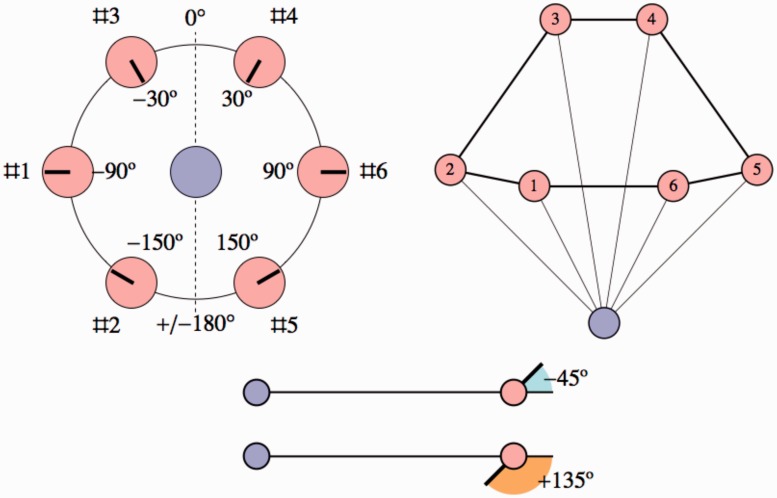
*Top left.* The azimuth is defined with respect to the forward direction defined in Figure 1 left, angles being counted in the clockwise direction (in this figure, all ranges have been set to the same value). *Top right.* This is a sketch of how the configuration appears to most observers, except that the ranges and azimuths have been set to their veridical values. *Bottom.* Gaze directions are reckoned with respect to the local direction of view, thus they are zero for actors in the dorsal, $\pm 180^{\circ }$ for those in the ventral pose. Here we show two example poses, one for negative and one for positive gaze angle.

The geometry of the experiment is sufficiently complex that it may be confusing for most, thus we clarify some definitions. The reader is invited to use Figure 5 as a reference.

The figure at top left shows the definition of the azimuth, the figure at top right shows an idealised response in which the azimuths and distance ratios are veridical and the figure at bottom shows the definition of the gaze angles. In the figure at top right we also indicate indices for the locations, which often come in handy in discussion.

Just for exercise, in the figure at top left, the gaze angles for Indexes 1 to 6 are 0deg,+90deg,±180deg,±180deg,-90deg and 0deg. Of course, the angles ±180deg are equivalent.

### Design of the Experiment

**Figure 6.**
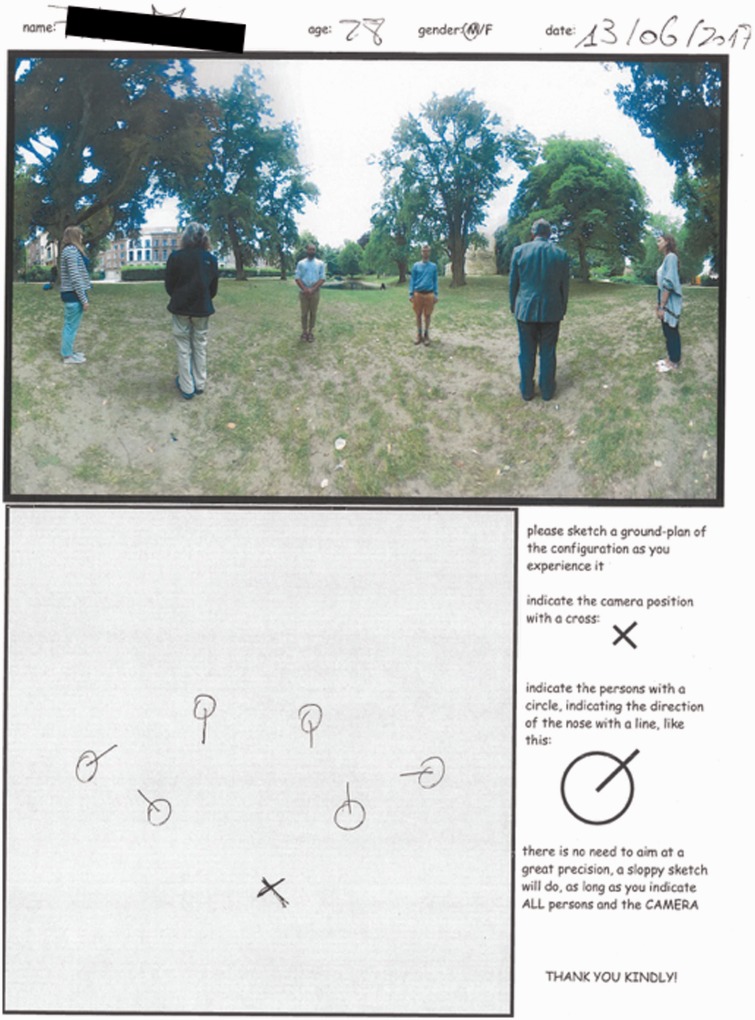
A sheet as used in the experiment. At top the stimulus and at bottom right the instructions. At bottom left the drawing area (which blank at the start of a trial). Here we show a typical drawing, this is the ‘response’. (All responses made available as a movie on the publisher's website). Notice that the method is self-documenting.

In the final experiment, observers were handed a piece of A4 paper (see Figure 6) in portrait orientation with the Mercator map as shown in Figure 4 printed in the upper half. They could fill in their personal data (name, age, gender and date) on a line at top. The lower half had an outlined, square, empty area with the instructions in the right margin. No further instruction was provided.

All analysis was done on the basis of the drawings, a typical one being shown in the drawing area of Figure 6. These drawings were digitised by hand using a program specially written for the occasion. The centres of gravity of the person marks and the camera marks were judged by eye.

### Observers

Observers were students and staff at the institutes of experimental psychology of the universities of Leuven and Giessen. Median age was 32 and interquartile range was 27 to 37. The gender ratio was 39% female. A total of 61 persons participated in the experiment, 24 from Leuven and 37 from Giessen.

## Observations

Participants had no conceptual problems with the task. As expected, no one asked what the field of view was. (If anyone had done so we would have offered that information.) Problems that occasionally occurred were due to the familiar fact that people often underestimate the final size of their drawing, thus finding themselves short of free space at some phase in the process. When the camera was drawn outside the frame, this was considered acceptable.

In cases where they inquired in retrospect whether they were ‘right’, they often had trouble to make sense of the physical ground plan and to relate it to the picture. Even after ‘knowing the solution’, no one could intuitively ‘see’ it. Quite a few even failed to understand the ‘solution’ at all. This lack of ability to ‘see’ the actual configuration also holds true for the authors: Although we obviously know exactly what the actual scene was like, we can only relate that to the picture in reflective thought, using geometrical and logical reasoning, we cannot spontaneously *see* it. In that respect it is similar to the classical geometrical illusions, where knowing the actual geometry does in no way help to get rid of the illusion.

The drawings were digitised into ordered lists of Cartesian coordinate pairs. All subsequent analysis was done on these data.^2^

## Analysis

Various types of analysis can be done on this data. However, we are careful not to overanalyse the data in view of the fact that participants delivered quick sloppy drawings and did not use any drawing or measuring instruments.

### Initial Categorical Checks

At an initial stage we removed four items from the list because obviously they were incoherent. In one case this was known to be due to language problems. Formal reasons were such factors as, for instance, a number of actors different from six, cases of all actors facing the camera, evidently in conflict with the pictorial content, or extreme long time to reflect on the response. This left 57 cases.

In 55 out of 57 cases (96.5%), the camera was located outside the convex hull of the actor locations. With a Bayes factor of 9.5 we have ‘substantial’ evidence (using Jeffreys prior and scale) for the fact that the observers located the camera at some distance in front of the group.

Another coarse check involves the nose directions. In 50 out of 57 cases (88%), all noses pointed into the interior of the convex hull. With a Bayes factor of 8.9 we have again ‘substantial’ evidence for the fact that all observers faced into the interior.

### Parameter Estimates

A very simple starting point is an overall average. First we computed the mean and covariance matrix for the six actor locations. The drawing was then translated, rotated and scaled to place the mean at the origin, the orientation of the largest variance horizontal, with the largest variance equal to 1. This brings all results into a common format. This is a necessary stage because observers used different placements, orientations and sizes in their drawings.

**Figure 7.**
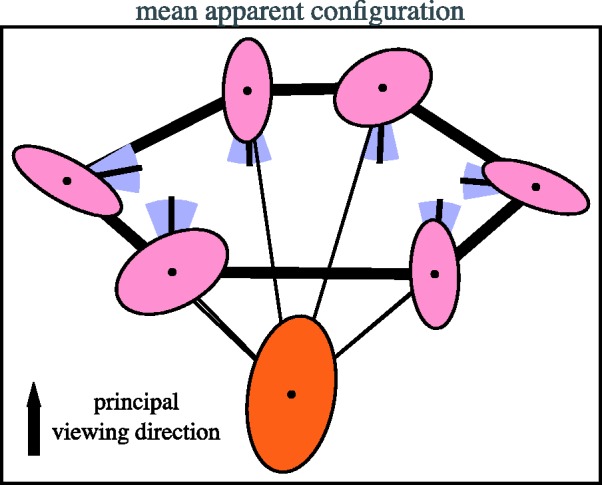
The normalized (see text) and averaged configuration. The camera position is indicated in orange, the actors in pink and the nose directions in blue. The thick black line suggests the spatial configuration of the group, the thin black lines the directions of the actors as seen from the camera location.

We then averaged over the full group, using circular statistics for the nose directions. In Figure 7 we show the result. The ellipses for the locations and sectors for the directions have been drawn at one standard deviation.

The result is encouraging, for it shows that the normalised data is quite homogeneous. This result is perhaps even better than expected.

#### The apparent scope

**Figure 8.**
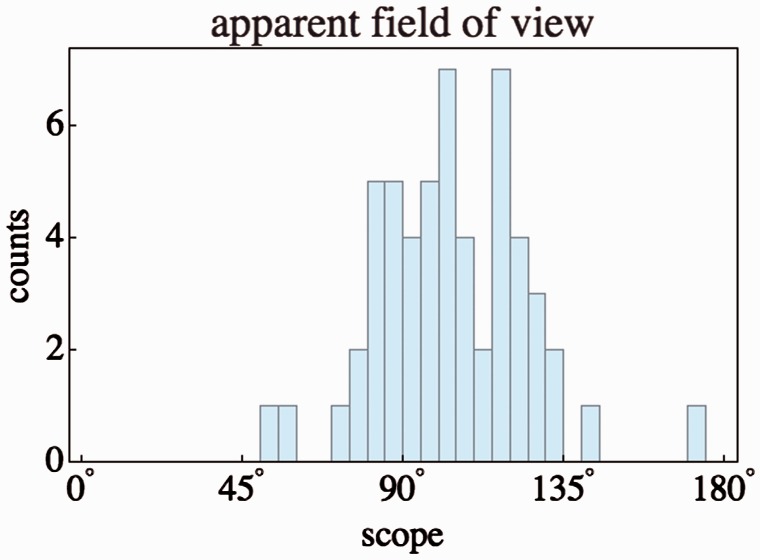
Histogram of the apparent scope.

One simple measure is the apparent angular scope of the configuration as subtended by the group seen from the camera location. The median is 104°, with an interquartile interval of 89° to 119°. The histogram is shown in Figure 8.

With a median wider than a right angle, the scopes are quite wide. However, it is evident that the scope is certainly less than 180°. Thus, virtually all participants saw the picture as pictorial content ‘in front of them’ or perhaps ‘behind the picture plane’. This is a major qualitative result.

#### Aspect ratio

For this initial pass through the data we defined the ‘aspect ratio’ as the square root of the ratio of the eigenvalues of the covariance matrix of the positions in the normalised data. The eigenvectors are almost all (exceptions noted later) in the anterior–posterior and left–right orientations, so this is a reasonable estimate in most cases.

The majority of the configurations have an aspect ratio quite different from 1. In all cases, the elongated hexagon is oriented with its major axis at right angles to the principal direction of view (see Figure 7). The configurations are extended in the lateral orientation or – equivalently – flattened in the (frontal) depth direction.

**Figure 9.**
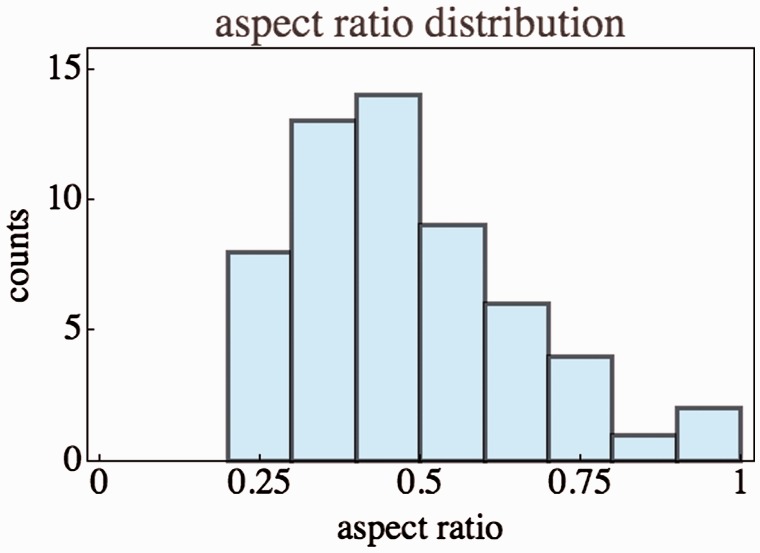
Histogram of the aspect ratios of the hexagonal group.

The median aspect ratio is 0.46 and the interquartile range is 0.34 to 0.59. In Figure 9 we show a histogram of the distribution. (In the discussion we have occasion to address the aspect ratios from another perspective.)

#### Depth ratios

**Figure 10.**
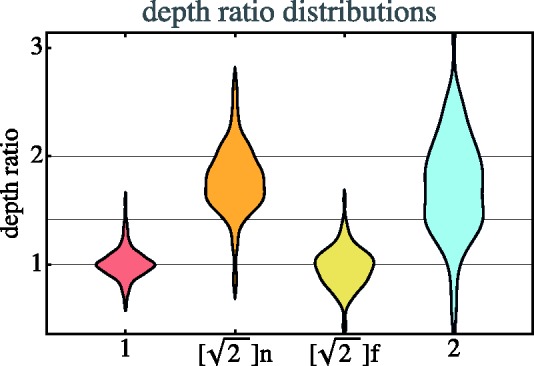
Distributions of depth ratios. Notice that the cases of $\sqrt{2}$ range ratios have been split into near (‘[$\sqrt{2}$]n’) and far (‘[$\sqrt{2}$]f’) instances. The 1, $\sqrt{2}$ and 2 levels have been indicated with the horizontal lines.

The range ratios (camera to actors) are either 1, $\sqrt{2}\approx 1.4$ or 2. The depth ratios can easily be obtained from the observed geometry. Since the depth ratios can easily be estimated from the relative sizes of the actors in the stimulus, one perhaps expects to find them reflected in the results. In Figure 10 we show distribution data.

The unit range ratios are $\left\{d_{1}/d_{6},d_{2}/d_{5},d_{3}/d_{4}\right\}$ (indices defined in Figure 5) as well as their reciprocals. Thus the median is not too interesting, only the spread is. The interquartile range is 0.927 to 1.08, which may be partly due to the fact that the actors were not identical in height.

For the range ratios 2 we use $\left\{d_{3}/d_{1},d_{4}/d_{6}\right\}$. The median is 1.76 and the interquartile range is 1.42 to 2.07, so these depth ratios are about 10% less than veridical.

The range ratios square root of 2 show a bimodal distribution. Here we need to distinguish between the near and the far range. In the near range we consider the ratios $\left\{d_{2}/d_{1},d_{5}/d_{6}\right\}$ and in the far range, $\left\{d_{3}/d_{2},d_{4}/d_{5}\right\}$. In the near range, the median ratio is 1.77 and the interquartile range is 1.609 to 1.96, and in the far range, the median ratio is 0.983 and the interquartile range is 0.852 to 1.09. So, the near and far ranges are indeed very different and both far from veridical, though in different directions.

Thus the results for the $\sqrt{2}$ range ratios are somewhat puzzling. After all, the veridical values are simply implied by a ratio of two linear stretches in the picture plane. Participants apparently do not use the optical size–distance relation to their best advantage, whereas it would be natural for them to assume that the actors were of (very roughly) similar height.

#### Apparent azimuths

The azimuths at which the camera sees the actors are spaced at 60deg intervals. This is reflected by a uniform spacing in the Mercator projection, even in the case of the left-most and right-most actors if one remembers the periodic topology of the horizon. One might expect the observers to space the apparent azimuths equally since they are fully displayed in the picture plane. This turns out to be hardly the case though.

**Figure 11.**
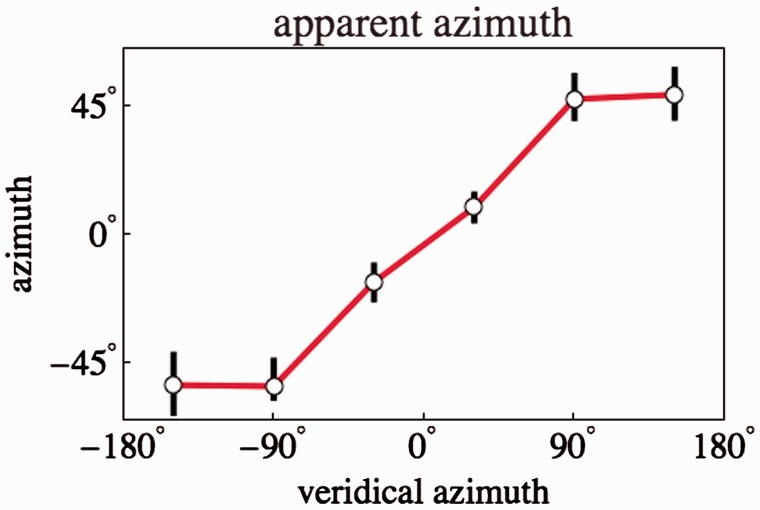
A plot of the observed azimuths against the veridical ones (Remember that $\pm 180^{\circ }$ are the same point on the horizon!). The azimuths of the outermost actors appear highly compressed.

Figure 11 shows a plot of the observed azimuths against the veridical ones. Apparently, the observed azimuths of the outermost actors are highly compressed. This is already evident from the average plot (Figure 7).

#### Nose directions

The veridical nose directions have been listed earlier, they are:

**Table:** 

Azimuth	Gaze
-150deg	+90deg
-90deg	0deg
-30deg	-180deg
+30deg	+180deg
+90deg	0deg
+150deg	-90deg

**Figure 12.**
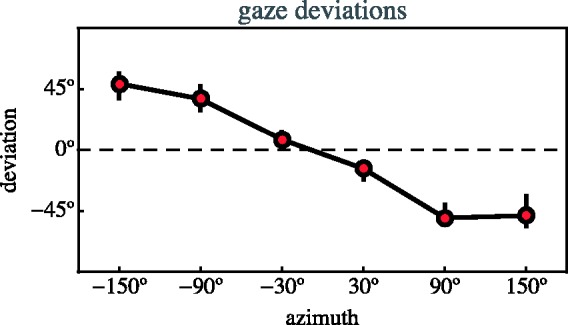
The deviations from veridical of the observed nose directions, plotted against the azimuth. The dots are medians, the interquartile range is also indicated, though hardly apparent.

The deviations (median observed nose direction minus the veridical direction) have been plotted against azimuth in Figure 12. (The figure also shows the interquartile ranges.) Clearly, the observations differ appreciably from veridical.

## Discussion

First some special cases are discussed and a discussion on generic results follows. Although the special cases get some attention here, one should not forget that the bulk of the data is best represented by the averages shown in Figure 7. The special cases form only a small minority. That is not to say they are not of major interest though. Of the two interpretations suggested in Figure 1 right (a fuller discussion follows later) one has the viewpoint *interior* to the circle of actors and the other *exterior* to that circle. (Read ‘in the round’, or something like that for ‘circle’ here, we obviously do not intend a perfect geometrical circle.) Both are quite reasonable interpretations. Whereas it is indeed very interesting to note that the large majority voted ‘exterior’, it is highly relevant that some voted ‘interior’: It shows that participants *have a choice*.^3^ A formal analysis also reveals two categorically different but optically equivalent (Ittleson, 1952) interpretations. Thus, it is a notable fact that generic observers have a very pronounced preference for one of these.

### Inside the Magic Circle

**Figure 13.**
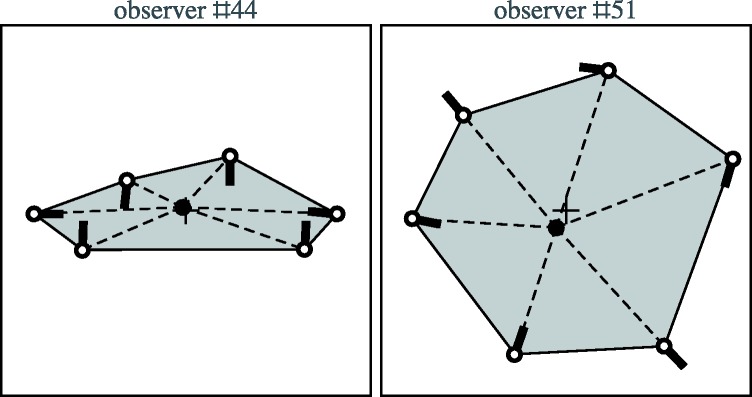
The two observers who entered the magic circle. Locations and directions of the nose are indicated by the white dots with thick black line elements. The cross denotes the origin of the (normalised) coordinates. The black dot indicates the camera location.

There were two observers who entered the magic circle (see Figure 13). These are mutually very different cases.

In the case of Observer 44 we see a standard configuration *except* for the location of the camera. Here the view is from outside the magic circle, whereas the camera has been placed inside. Of course, this latter placement is fully inconsistent with the configuration. The fact that two actors evidently had their back turned towards the camera is completely ignored. This drawing is possibly a mixture of what the observer actually saw and some aspect of what the observer knew about 360deg cameras but could not integrate with the percept.

The case of Observer 51 is more interesting. This was in fact the only observer that entered the magic circle in a qualitatively consistent manner, the inconsistencies (which are not at all minor) being of a quantitative nature. This observer marked the actors both in the stimulus and in the drawing, so we can be certain that the two noses pointing outwards belong to the actors seen from the dorsal side and that the noses pointing at right angles to the visual direction belong to the actors seen in profile. The responses are indeed quite consistent, except for the fact that the distance ratios have been fully ignored. Apparently the urge to see a ‘circle’ was much stronger than the additional visual evidence.

There was actually another observer among the ones left out from the data set who started with drawing a circle, putting the camera at the centre, the actors in a regular hexagon – fully ignoring angular size and horizon dip cues. The observer then rounded this off by putting the nose directions as would fit a view from far out of the magic circle. In this case the drawing was evidently made on the basis of guesses in reflective thought as was also evident from the time taken to reflect (the reason for the decision to ignore this response).

### Convexity

**Figure 14.**
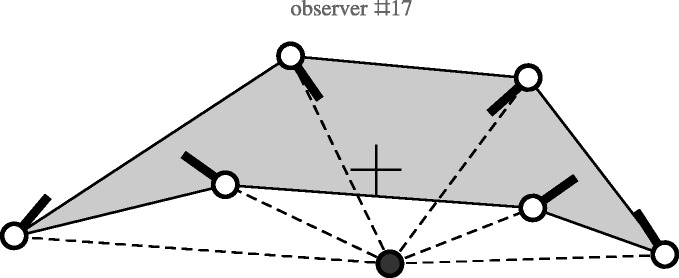
The response of Observer 17. Locations and directions of the nose are indicated by the white dots with thick black line elements. The cross denotes the origin of the (normalised) coordinates. The black dot indicates the camera location.

There are three nonconvex responses, two of these trivial, because of minor sloppiness in drawing, the remaining one is interesting and merits mention (Figure 14).

The configuration is evidently nonconvex and the scope is unusually large, about 180deg. This is the only observer that noticed the huge scope, although still not entering the magic circle. The response shows the observer to notice the nose direction with respect to the visual direction, no other observer did that. Also the depth ratios are reasonably well reflected in the response (compare Figure 10). All in all, this observer picked up more cues than almost anyone else. The response is close to possible interpretations that include ‘the space behind the back’ (see later).

We do not discuss the convexity issue further at this point, since it will be more fully treated in terms of the log-polar model introduced later. (A configuration that is nonconvex in physical space may well be convex in visual space.)

### The Overall Qualitative Outcome

The major result of the experiment is neatly summarised in the average results depicted in Figure 7. Apparently the actors are perceived as arranged in a slightly flattened hexagon, its long axis perpendicular to the principal direction of view (defined in Figure 7). The camera is located *outside* the hexagon. All nose directions point into the interior of the hexagon. Thus the experience is of the magic circle *as seen from the outside*.

Such a result is not unexpected (Koenderink & van Doorn, 2017, in press). The impression cannot even be said to be ‘non-veridical’ in the sense that the picture itself is not a touchstone for veridicality. The result is due to these major factors:

^ observers implicitly assume some finite scope, in many cases related to the extent of their apparent field of view (Koenderink, van Doorn, & Todd 2009). The latter is almost invariably smaller (usually much smaller) than a half-space;
^ observers relate the apparent spatial attitude of pictorial objects to their local (apparent) visual directions (Koenderink, van Doorn et al., 2010);
^ observers tend to apply templates in favor of ‘inverse optics’ algorithms (Koenderink, 2011).

The same factors give rise to huge errors in regular wide-angle photographs, as we have documented in the past (Koenderink, van Doorn et al., 2010).

Next we discuss these issues in more detail in terms of the log-polar model of visual space (Koenderink & van Doorn, 2008).

### Discussion of Selected Quantitative Details

In this subsection we discuss a number of remarkable regularities in the data.

#### Azimuth pattern

One pictorial structure that might be thought to be obvious to all observers because immediately visually present is the pattern of azimuths. Even without fully parsing the stimulus the regular spacing of the major pictorial objects (the actors) is evident. One might expect that basic fact to be reflected in the responses. Perhaps remarkably, it is not, as is evident from Figure 11.

This might suggest that observers generally put the camera too close to the hexagonal configuration. This is indeed in accord with the generally wider than expected scopes.

One may speculate that it perhaps has to do with the limited drawing area. Of course, there is no way to check for that in the data, it would imply another (major) effort to address the issue empirically.

#### Depth ratios

The distributions of depth ratios as plotted in Figure 10 gives rise to some concern.

The depth ratios 1 are responded to veridically, with little spread. This is no more than expected. The depth ratios 2 are underestimated, although only by about 10%, also, not much reason for concern. Their spread is large.

The depth ratios $\sqrt{2}$ are wildly off the mark though. Moreover, the distributions are markedly bimodal with the ratios in the near ranges being treated quite differently from those in the far range. Those in the near range are overestimated by about 30%, whereas those in the far range are underestimated by about the same amount. In the far case the spread is moderate, in the near case rather larger.

**Figure 15.**
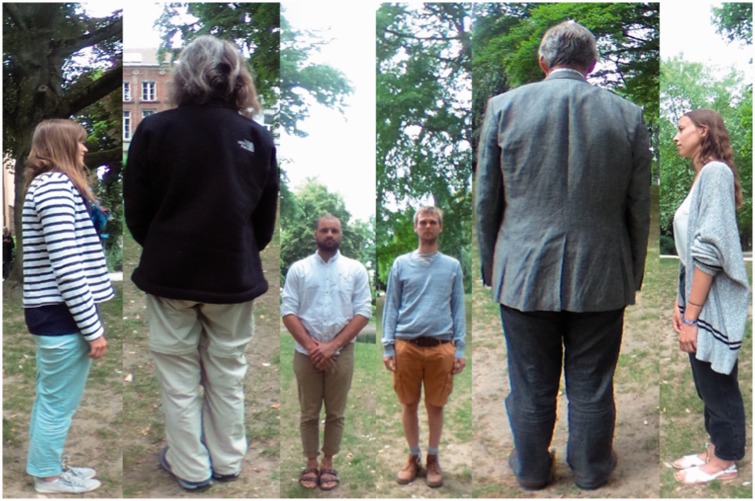
These are cutouts from an equirectangular map, thus the vertical dimension is simply the elevation *ɛ*. For practical purposes, the height ratios in the Mercator projection are quit close. It is evident that we have ratios 1, 1.4 and 2.

Such effects are surprising in view of the clear pictorial cues. As seen in Figure 15 the pictorial evidence leaves little doubt as to the depth ratios. Observers are expected to use such cues fully automatically. There is something going on here that is hard to put the finger on, at least on the basis of the present data. Again, it would imply another (major) effort to address the issue empirically.

#### Aspect ratios and scopes

The cases of the aspect ratios and the scopes are discussed together since they appear mutually related.

The apparent scopes appear rather larger than expected. The median corresponds to a focal length of about 14 mm on a 35-mm camera (the classical 24 × 36 mm Leica format), generally considered a fairly wide wide-angle (the shortest lens for the Leica is the 12 mm Heliar produced by Voigtlander). Of course, it is still fully located in the frontal half-space of the (imagined) camera.

From an earlier investigation (Koenderink et al., 2009) we know that the median ‘apparent field of view’ (perhaps the ‘diameter of the visual field’) is about a right angle (as Helmholtz reports for his own subjective feeling) with a very wide distribution from about 10deg to over 210°. In the latter case, people feel to ‘see behind their ears’, in the former, they feel ‘everything to be in front’ of them.

The fact that people are not comfortable with a limited drawing surface may have played a role, in that they perhaps place the camera closer to the configuration than they would have if given more drawing space. (Of course, being confronted with an essentially unlimited surface might intimidate them even more, there is no obvious way to handle such inhibitions.) One can only speculate.

**Figure 16.**
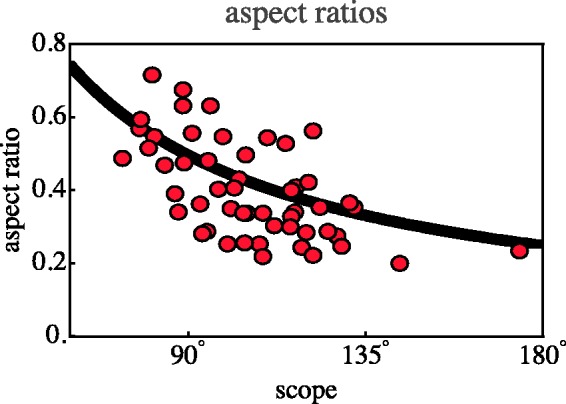
Scatterplot of the aspect ratios against the scopes (red points). The curve is the theoretical prediction, the points are aspect ratios as measured in the log-polar visual space model. Although the spread is large, as was to be expected, the prediction is evidently in the right ballpark. (Two cases of interior views left out.)

Here we reconsider the issue of aspect ratios. In Figure 9 we measured aspect ratios in the drawing. It is probably more useful to consider these in the log-polar representation (see next section). So we compute ‘aspect ratio’ as measured in log-polar visual space. These aspect ratios also have a large spread, in this case with an interquartile range of almost 0.3 to 0.5. On theoretical grounds one expects a relation between the scope and the aspect ratio (see next section). This can be tested via a scatterplot involving all cases (Figure 16). This graph should be interpreted in terms of the log-polar model introduced later.

#### Apparent spatial attitudes

The nose directions with respect to the local camera-viewing-direction are immediately given as pictorial evidence. Actors are clearly seen either in anterior or posterior frontal attitude, or in left or right profile.

However, observers apparently distinguish between various directional frames. In this case they show systematic deviations from what appears to be pictorially given, as documented in Figure 12.

We believe such systematic effects to be due to the same factors we have studied in some detail earlier (Koenderink, van Doorn et al., 2010), so it may be said that they meet our expectations.

### The Log-Polar Model of Visual Space

For simplicity we only consider the horizontal plane at eye-height, thus, if we say ‘visual space’ (usually a hemisphere augmented with depth), we limit the discussion to the horizon augmented with depth. What is ‘optically specified’ are just visual directions, the depths are added in the psychogenesis of visual awareness.

In a model of visual space, one has to address the issue of depth values. The best known example is ‘inverted optics’: one simply computes the depths from the optical data (Marr, 1982; Poggio, Torre, & Koch, 1985), if this does not work one guesstimates them (Knill & Richards, 1996). This appears reasonable enough, but what if none of this works to satisfaction? Is it possible to say something about the structure of visual space anyway? Here is a kind of poor man’s inference, based on Euclid’s Optics (Burton, 1945):

^ in the absence of specific knowledge, all visual directions are equivalent, none is preferred. Hence the structure of visual space should be *invariant with respect to angular translations* (rotations about the nadir-zenith axis).
^ in the absence of specific knowledge, there is no preferred ‘unit of distance’. Hence the structure of visual space should be *invariant with respect to distance scalings* (dilations or contractions about the view point).

**Figure 17.**
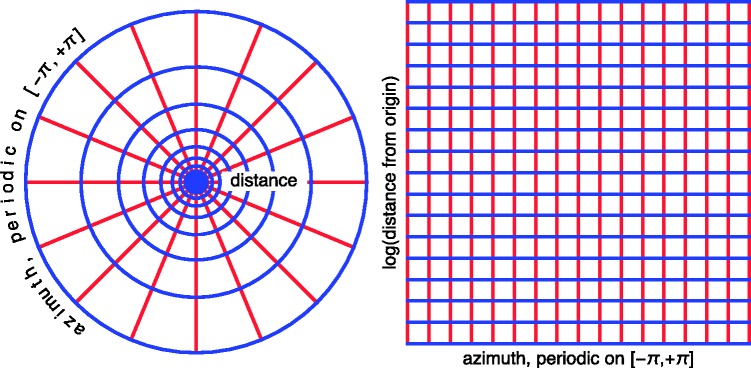
The ‘log-polar’ model of visual space. At left, a polar coordinate system in the Euclidean plane. The system of ‘rays’ is obviously invariant with respect to rotations about the origin. The radii of the system of circles concentric with the origin have been distributed such that the system is invariant with respect to uniform scalings about the origin. Thus, the whole system is invariant with respect to rotations and scalings. This models the structure of optical information as discussed by Euclid in his book on optics. At right, this configuration has been transformed by the log-polar map. One obtains a Cartesian grid, small regions of the grid are geometrically similar to the corresponding region in physical space. The map is conformal. Arbitrary translations of figures in log-polar space derive from optically equivalent figures in physical space. This model has been shown to account quite well for observer responses with very wide fields of view.

These very basic invariances imply a minimal structure of visual space. It can be formalised in various ways. A very simple formalisation is the log-polar model (see Figure 17).

Use azimuth ϕ (angle from the anterior direction, ranging from -π (or -180deg) to +π (or +180deg)) and the logarithm of the distance from the origin ξ=logϱ (ϱ the distance from the origin in an arbitrary unit) as Cartesian coordinates. Thus *ξ* ranges from -∞ (where the eye is) to +∞ (the far distance). The invariances imply that arbitrary translations in the ϕξ–coordinate system are irrelevant.

This model has many nice properties, for instance, it is conformal, thus has no deformations of local details.

**Figure 18.**
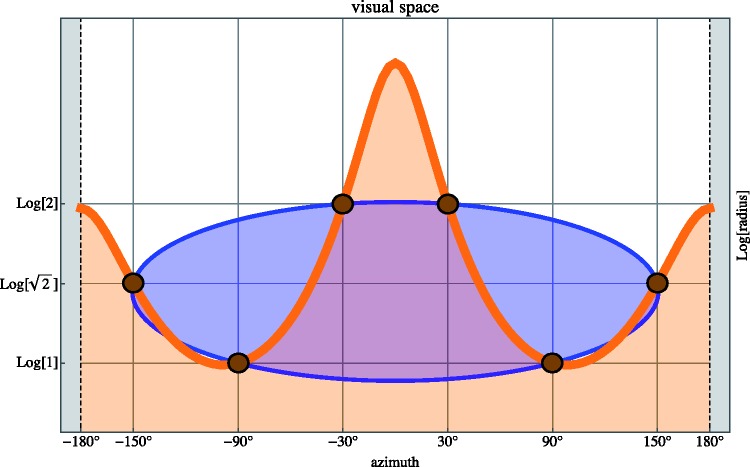
The configuration shown in Figure 1 *right* plotted in visual space according to the log-polar model. Now the strange blue banana shape is a perfect ellipse, whereas the orange ellipse is the orange area which extents all the way to -∞. It is also *closed* (although it may not look that way), because the left and right edges of the plot are actually the same visual ray, in the posterior direction.

As a first application we plot the map of the two regions plotted in Figure 1 right in visual space (see Figure 18). Now it is immediately obvious why the strange blue banana shaped region in Figure 1 is of interest: It is the unique ellipse that passes through the points of the stimulus hexagon in visual space. It is perhaps more difficult to see that the orange region is bounded by a closed curve too, but notice that the left and right vertical boundaries are actually *the same visual direction*. Remember that ‘the eye’ is at {0,-∞}. In the log-polar model the eye is outside the plane, because *the eye cannot see itself.* It is of considerable interest to compare the complementary depictions of *the same areas* in Figures 1 right and 18.^4^

Starting from this model picture, we may attempt a bold extrapolation: What if we *scale the azimuth*?

Scalings of the depth dimensions are reasonably well understood. We have worked on that for many years. Scalings of the azimuth dimension have – to the best of our knowledge – not been considered before, except in the (very special and limited) case of linear perspective (Pirenne, 1971). Scalings of the azimuth dimension are of immediate importance in the perception of photographs for which the scope is essentially unknown.

**Figure 19.**
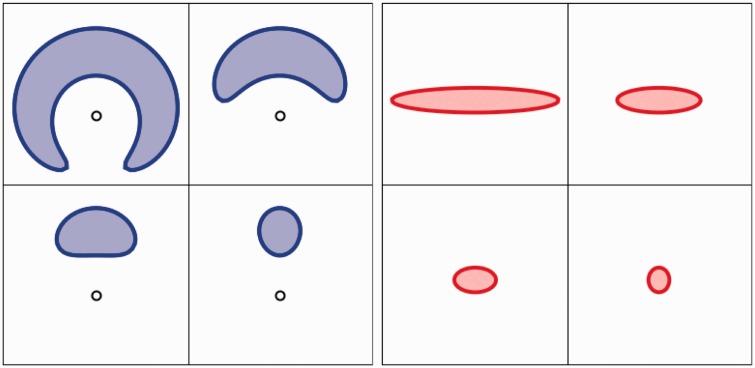
Here are examples of *equivalent* configurations in physical space (left) and visual space (right). *Left:* The configuration at top left corresponds to the ‘true sized’ configuration in physical space (Figure 1 right). The other three cases involve scalings of the scope, by factors of 2, 4 and 8. Notice that for the factor 4, the shape is ‘almost’ convex, the change to convex happens at a factor of $(\sqrt{\sqrt{14}}/\sqrt{\text{log}8})\pi \approx 4.124\ldots$. For larger factors, the banana-shape gives rise to an ovoïdal convex region as in the case at bottom right. In these figures, the eye is indicated with the open dot. *Right*: The same four configurations in log-polar space. These are all perfect ellipses, they have been scaled in scope, that is the horizontal dimension, and the vertical dimension is the same for all. Thus, this is a anisotropic scaling in visual space. In these figures, the eye is at {0,-∞} (far down the page!). The scaling allows a configuration that extends behind the observer (left figure at top-left), to be confined to the frontal region (the other three figures at left). Such scalings of the apparent scope appear quite natural when observers are not aware of the true scope of a picture.

Simply scaling scope we obtain the interpretations shown in Figure 19. Of course, the crucial application is to scale from the space behind your back (or, equivalently, in front of the picture place) to the space in front of you (or, equivalently, behind the picture place). Here it is already quite clear from all kinds of data that *visually, there is no space behind your back* and that it is *in bad taste, and almost impossible to pull off, to show objects in front of the picture plane*.^5^

Thus it is extremely interesting to notice that the (trivially simple) log-polar model does quite a good job to post-dict the present data. The exterior view model fits the data quite well. In the aspect ratio plot (Figure 16), the aspect ratios (square root of ratio of eigenvalues of the covariance matrix of the locations in visual space) are evidently well fitted. Thus one may actually extrapolate from azimuths behind the camera to azimuths in front of the viewer (or behind the picture plane), as demonstrated in Figure 19. (The corresponding figures for the categorically different case of Observer 17 are Figure 1-right (physical space) and Figure 18 (visual space), the bluish-tinted cases.)

In visual space (Figure 19 right) the scaling transforms an ellipse into another ellipse, thus the plot of Figure 16, where the aspect ratios have been measured in visual space is very ‘natural’. The definition of ‘aspect ratio’ in physical space (although formally fine) seems more contrived.

In the past we have already shown that pictorial space allows for huge (anisotropic) scalings in the ξ=logϱ–domain (Koenderink, van Doorn, & Kappers, 1994). Apparently any anisotropic scaling with principal axes along the ϕξ–dimensions can be admitted. That such an anisotropic scaling works so well is not something that could have been expected, it is a major result that significantly extends our previous conclusions.

We know of no other data addressing this issue. It is evidently of great interest to attempt to collect more though.

## Conclusions

Participants were confronted with a postcard-like photograph, obviously of a natural scene, without any information concerning the scope of the field of view captured by the camera. This was intentional as it is closest to generic applications. Almost no newspaper, magazine or textbook prints the width of the field of view in the legends (often there is no legend to begin with).

Although an obvious fact, almost irrelevant to relate, this goes *squarely against the grain of conventional understanding of correct depiction*. The conventional theory of correct depiction is solidly based on linear perspective or, more generally, on the conservation of angular relations between visual directions (Pirenne, 1971). On the one hand, it is generally (but silently!) understood that pictures will ‘work’, no matter what, on the other hand, it is a universally agreed fact that visual directions should be conserved. There is obviously some uneasiness here, in that many ‘wrong’ depictions often still give rise to ‘normal’ impressions. To look ‘wrong’, one typically needs to violate topological relations, as pioneered by artists like Picasso in the early 20th century (Berger, 1989).

The familiar Ames room (Ittleson, 1952) still manages to induce wonder, yet it works *because* it conserves visual directions. Ames demonstrated this loud and clear, although the significance of his message tends to be underestimated. He demonstrated that pictures are *infinitely ambiguous*, something that few scientists are ready to hear even today. For instance, it implies that ‘inverse optics’ per se is impossible. But in viewing and (hopefully) understanding pictures, the public routinely goes way beyond Ames. The Ames demonstration is trivial, but, because of that, important.

The really interesting cases involve deformations of the bundle of visual rays (Burton, 1945).

In this study we move away from the Ames case in that we do not conserve visual directions. It is perhaps of some interest though, because it involves the space behind the back, or in front of the picture plane. Our empirical results at least suggest that a fairly simple model suffices to get at least the basics right. *Why* such a simple model would work is something we are not ready to comment on. We are not aware of any argument from contemporary brain science that might conceivably illuminate this matter.

An interesting aside is that people who are (or have been made) intellectually aware of the actual physical layout of the scene are generally (we met no exceptions) still unable to ‘see’ this in terms of their visual awareness. A different approach would have been to tell the participants up front what they will be confronted with. Perhaps, it would not be too hard to instruct participants so as to arrive at veridical judgements through a mixture of geometrical and logical reasoning. It does not work anyway. Even when people know what is there, they stubbornly see what they see at first blush. Awareness is not a cognitive judgement. Visual artists trust their eyes, ignoring their reflective thoughts.

**Figure 20.**
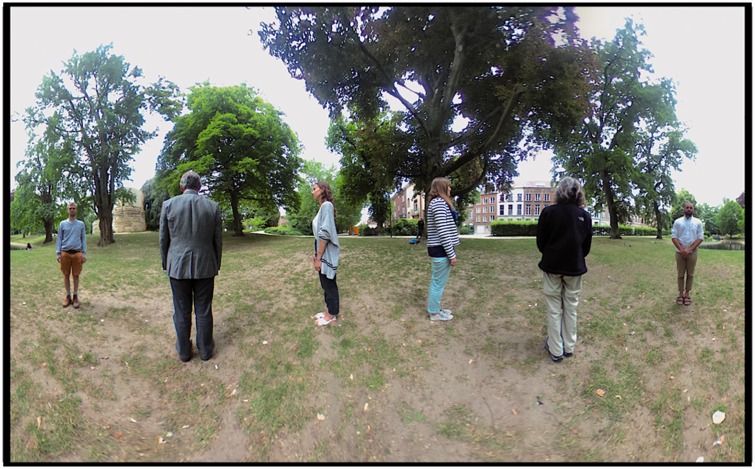
A picture of the scene after a $180^{\circ }$ rotation of the frontal direction. Notice that the actors seen in profile are actually adjacent to each other. The configuration now appears nonconvex, a bit like looking at the convex side of a banana.

Why the difficulties? We speculate that this has to do with the fact that there is no visual space behind one’s back (Phillips & Voshell, 2009). As a consequence, pictures are intuitively understood to only show part of what could be in front of one. Closely related to this is that the two actors seen in profile in the stimulus are adjacent to each other in the physical scene (see Figure 20, compare Figure 1), yet look to be at maximum separation in the picture. The shortest connection between them would have to cross the posterior meridian of the optic array, an area behind one’s back. In the picture, this connection would have to go by way of the left and right edges of the picture, which actually should be identified, again something that is not possible if everything is experienced as in front of the observer (Koenderink & van Doorn, 2017).

An actual picture of everything in front of the observer only shows part of the configuration (Figure 3). The depicted part is seen as essentially veridical.

**Figure 21.**
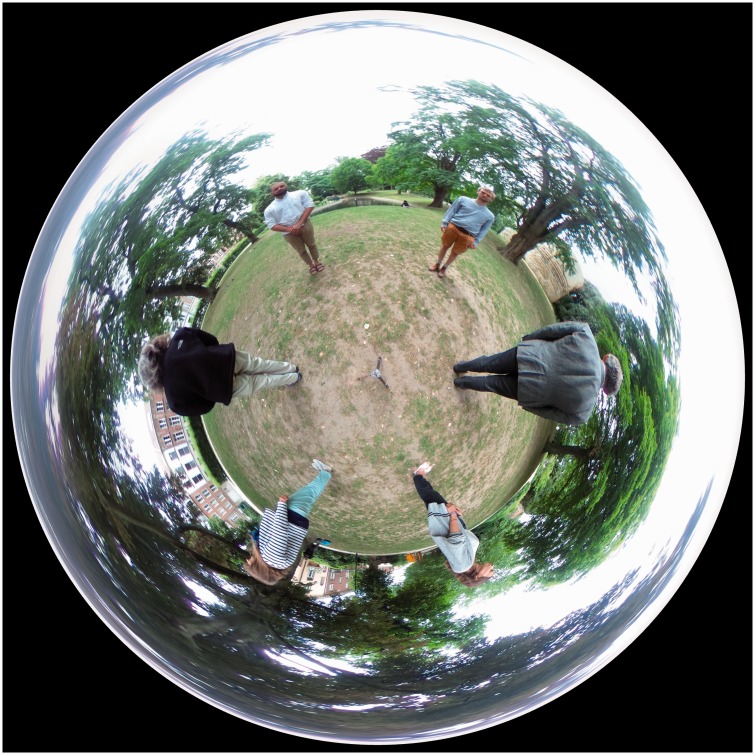
A Postel map based on Riemann normal coordinates centred at the nadir. The whole circular circumference represents the zenith, a single point. This map is neither conformal nor area true. It is quite nice near the origin (the nadir) though, essentially up to the horizon. Distances from the nadir and angles at the nadir are perfectly represented. Notice that the tripod on which the camera was mounted is visible at the centre. The camera cannot see itself. It takes two 190° back-to-back fish-eye photographs and stitches these together in the camera. The output is an equirectangular representation of the full optic array.

A more or less veridical view of the whole scene can perhaps only (we are not aware of alternatives) be pictured in a bird eye’s view of the scene (Figure 21). This is a Riemann normal coordinates map (also known as Postel’s projection (Flocon & Barre, 1968)) centred at the nadir. In this map, the full circular picture frame represents the zenith, that is a single point, thus may have some counterintuitive properties. Such renderings have recently become popular as ‘small planet’ images. Indeed, the horizon is a circle, and the full viewing sphere is mapped inside the disk.

The results from the analysis are clear enough. Virtually all participants are aware of a scene that fits the space in front of them, with the view point outside of the configuration. The relevant pictorial cues available to the observer are the horizontal separations, the heights in the picture plane, the relative heights of the actors and the apparent spatial attitudes with respect to the local visual direction (frontal, posterior or profile view, in the latter case facing left or right). The angular extent of the configuration is not indicated, thus it has to be *assumed* by the observer. It will necessarily be idiosyncratic. From previous experience with picture perception one may expect values to lie about the right angle, albeit with a huge spread. This is indeed what we find.

In any case, the results are clear evidence for the fact that the magic circle is almost impenetrable, only 1 of our 61 participants spontaneously succeeding. As we have shown in a related study, the same holds for the magic sphere – the viewing sphere (Koenderink & van Doorn, in press). For the latter case historical evidence for an intellectual awareness of this topo-agnosia existed for centuries (Goldstein & Hon, 2007; Stevenson, 1921). We are not aware of such evidence relating to the magical circle though.

By way of conclusion, it is easily possible to design configurations that will be interpreted by virtually all observers in some intended nonveridical manner. Since photographs (other than drawings or paintings) tend to be taken for optical truth, this may well be of interest to movie directors and illustrators. It may also serve as a warning against ill-considered use of wide panoramic images for applications in which a roughly veridical impression is desirable. Think of the real estate business, travel agencies and so forth. It is perhaps especially important in the court room (Carter, 2010; Sholik, 2015). Whether straight-out-of-the-camera panoramic images might be admitted as legal evidence should be a serious issue. 

## Supplementary Material

Supplementary Material

## Notes

1 Define azimuth *α* and elevation *ɛ* similar to geographical longitude and latitude, where the zenith takes the place of the north pole, the nadir that of the south pole and the horizon that of the equator. Let the principal viewing direction be α=0deg,ɛ=0deg. In the Mercator projection, the meridians map on vertical straight lines (vertical coordinate *η* say), the latitude circles on horizontal straight lines (horizontal coordinate *ξ* say). One simply sets ξ=α. The mapping on the verticals is defined as $\eta ={\text{gd}}^{-1}(ɛ)$, where ${\text{gd}}^{-1}$ denotes the inverse Gudermannian function. The map can be shown to be conformal. The magnification is sec(ɛ) and thus explodes at the zenith and nadir. At ɛ=60deg, it is already twice that at the equator.

2 The full data set is available on the website of the publisher.

3 Indeed, reviewers of the manuscript suggested that the task was entirely trivial with only one possible outcome. We obviously disagree.

4 The outline of the orange area has a simple expression in physical space:

$$x^{2}-\frac{\sqrt{2}-1}{3}y(\sqrt{3}-y)=1$$

whereas the outline of the blue region has a simple expression in the log-polar domain:

$$\frac{9}{\pi^{2}}\phi^{2}+\frac{20}{(\text{log}2{)}^{2}}(\xi -\frac{9}{20}\text{log}2{)}^{2}=\frac{63}{10}.$$

Both represent perfect ellipses in their particular domain. In either case, the expressions in the other domain are hardly enlightening. The funny constants in these expressions are a consequence of the π/3 azimuthal spacing and the 1, $\sqrt{2}$ and 2 distances, which were assigned arbitrarily so as to make the configuration come out right.

5 There are numerous famous (or infamous) examples in the arts of Western linear perspective. For instance, consider the hand of Captain Frans Banninck Cocq and the spear of Willem van Ruytenburch bursting through the picture plane in Rembrandt’s *Nachtwacht*. Although often admired, it is a gimmick in doubtful taste.
